# Phenotypic Spectrum of Gastric Adenocarcinoma and Proximal Polyposis of the Stomach (GAPPS) in Denmark: A Case Series Characterizing the First Danish Families With the *APC* Promotor 1B Variant c.‐191T > C

**DOI:** 10.1155/crig/4024148

**Published:** 2026-01-08

**Authors:** P. A. Skat-Rørdam, A. M. Jelsig, J. G. Karstensen, A. H. Petersen, M. B. Madsen, T. D. Jensen

**Affiliations:** ^1^ Department of Clinical Genetics, Copenhagen University Hospital, Rigshospitalet, Denmark, gentoftehospital.dk; ^2^ Department of Clinical Medicine, University of Copenhagen, Copenhagen, Denmark, ku.dk; ^3^ Danish Polyposis Registry, Gastrounit, Copenhagen University Hospital–Amager and Hvidovre, Hvidovre, Denmark; ^4^ Department of Clinical Genetics, Vejle Hospital, Vejle, Denmark; ^5^ Department of Genomic Medicine, Copenhagen University Hospital, Rigshospitalet, Denmark, gentoftehospital.dk

**Keywords:** APC, GAPPS, gastric adenocarcinoma and proximal polyposis of the stomach, gastric cancer, gastric polyps, promotor region

## Abstract

Gastric adenocarcinoma and proximal polyposis of the stomach (GAPPS) is a rare autosomal dominantly inherited gastric cancer syndrome that is characterized by fundic gland polyposis of the stomach (> 100) and an increased risk of gastric cancer. The genetic cause is recognized as a pathogenic variant in the promotor 1B of the APC gene. Presently, there are no established clinical criteria, and current guidelines are based on limited evidence. In this report, we identified two families with GAPPS. Family I had a family history of gastric cancer, and we identified seven family members with GAPPS. The diagnosis was verified by endoscopic findings of polyposis and genetic analysis identifying a variant in the promotor 1B of the APC gene, NM_001127511.3: c.‐191T > C. In Family II, the same pathogenic variant, NM_001127511.3: c.‐191T > C, was detected as an incidental finding in a 61‐year‐old patient with hepatocellular carcinoma, clear cell renal carcinoma, and small cell lung cancer. An esophagogastroduodenoscopy (EGD) at the age of 59 had revealed only one small fundic polyp. This is the first report of patients with GAPPS from Denmark, and it emphasizes the variable phenotypic expression and subsequently the difficulty of surveillance and genetic counseling in these patients and their families.

## 1. Introduction

Gastric adenocarcinoma and proximal polyposis of the stomach (GAPPS) is a novel and rare gastric cancer syndrome first described by *Worthley* et al. in 2012 [1]. The syndrome is characterized by fundic gland polyposis of the stomach sparing the antrum (> 100 polyps) and a predisposition to gastric cancer. However, knowledge of the phenotypic spectrum is limited. The underlying genetic cause have been identified as single nucleotide variants in the promotor 1B region of the APC gene inherited in an autosomal dominant manner. A pathogenic variant in this region causes a reduced binding of the transcription factor Ying Yang 1 (YY1) and, hence, a reduced activity from the promotor 1B [2].

Fundic gland polyposis of the stomach is also seen in familial adenomatous polyposis (FAP), but as opposed to FAP, GAPPS presents with minimal to no polyposis of the duodenum and colon [3]. Due to the increased risk of gastric cancer, a rigorous gastroscopic surveillance program and, in some cases, prophylactic gastrectomy are indicated.

The prevalence is unknown but could be expected to increase as genetic diagnostics improve. To date, only about 110 patients with GAPPS have been identified worldwide. There have been reports of GAPPS from Australia, North America, Japan, the Czech Republic, and Spain [4]. Here, we present the first report of patients with GAPPS from Denmark, illustrating the variable phenotype.

## 2. Case Report

### 2.1. Family I

**Figure 1:**
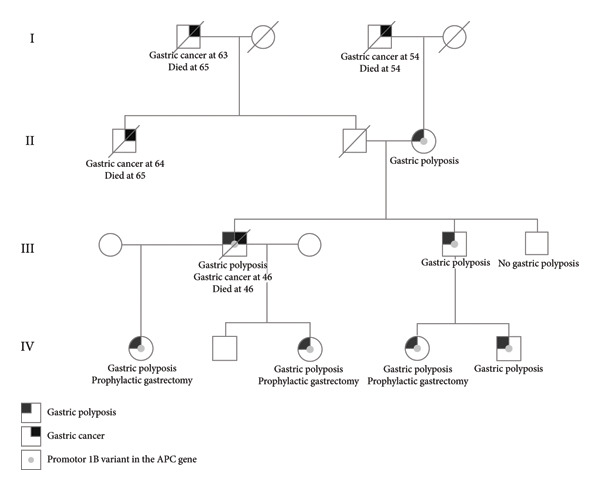
Pedigree of Family I.

Two brothers, aged 43 and 39 years, were referred to genetic counseling due to the recent loss of their brother to gastric cancer at 46 years of age. Both brothers had no significant medical history and no prior genetic counseling. The deceased brother had been diagnosed with disseminated low differentiated gastric adenocarcinoma and passed away shortly after. Esophagogastroduodenoscopy (EGD) had also revealed fundic gland polyposis with areas of dysplasia. There was a family history of gastric cancer (see Figure 1). The brothers’ paternal uncle was diagnosed with gastric cancer at the age of 64 and died at 65, their paternal grandfather was diagnosed with gastric cancer at the age of 63 and died at 65, and their maternal grandfather was diagnosed with gastric cancer at the age of 54 and died at 54. Unfortunately, no information regarding gastric polyposis in these family members were available. Their mother had previously been diagnosed with uterine cancer, sarcoma of the endometrium, colorectal cancer (CRC), and pulmonary cancer at ages 40, 60, 73, and 74 years, respectively.

An EGD of the youngest brother revealed no gastric polyposis, while the older brother had fundic gland polyposis with sparing of the antrum, initially without dysplasia but later progressing to low‐grade dysplasia. Initially, a restricted gene panel, which did not include the APC gene, was analyzed and revealed no pathogenic variants in either of the brothers. Later, a reanalysis of the data in an expanded panel, which included the APC gene, revealed a heterozygote pathogenic variant in the *APC* promotor 1B, NM_001127511.3: c.‐191T > C, in the deceased brother and the older brother, confirming the diagnosis of GAPPS. The youngest brother did not have the variant. Further genetic investigation revealed that the variant was inherited from the mother, who had fundic gland polyposis without dysplasia consistent with GAPPS. EGDs of the brothers’ children, who had inherited the variant, showed severe fundic gland polyposis. In two cases, there was low‐grade dysplasia, in one case, a smaller focus with low‐grade dysplasia, and in the last case, no dysplasia (see video 1). The two children with low‐grade dysplasia and the child with no dysplasia have since undergone prophylactic gastrectomy at ages 28, 28, and 37, respectively. The subsequent histopathological examination showed areas with low‐grade dysplasia in all three and one also had several foci with high‐grade dysplasia.

Colonoscopies were performed in all of the identified family members and revealed a total of four hyperplastic polyps, six tubular adenomas, and one sessile serrated lesion among three family members (see supporting information).

One of the family members underwent fertility treatment and preimplantation genetic testing selecting embryos without the *APC* promotor 1B variant, which has resulted in the birth of a healthy child.

### 2.2. Family II

A woman was referred to genetic counseling due to a medical history of hepatocellular carcinoma and clear cell renal carcinoma, diagnosed at the age of 58, and a recent diagnosis of small cell lung cancer (SCLC) at the age of 61. There was a family history of breast cancer, prostate cancer, and CRC on the maternal side of the family. She had two healthy grown‐up children. The patient previously had an EGD, at the age of 59 years, which was normal apart from the finding of one small fundic gland polyp with no dysplasia. A prior colonoscopy had also been performed, revealing one hyperplastic polyp and one tubular adenoma. She received genetic testing using a cancer gene panel comprising 46 genes, including the APC gene, which revealed a heterozygote pathogenic variant in the promotor 1B of the APC gene, NM_001127511.3: c.‐191T > C. Clinically, she did not have any known manifestations of GAPPS. Unfortunately, her condition deteriorated, and she died shortly after. Therefore, no additional examinations were performed. The family was referred to genetic counseling.

## 3. Discussion

We present the first eight patients identified with GAPPS in Denmark. All patients had the same *APC* promotor 1B variant NM_001127511.3: c.‐191T > C, which has been reported previously [1, 2, 5–10]. We observed a significant difference in the severity of symptoms. The endoscopic findings in Family I were consistent with those previously reported for GAPPS, but in Family II, the patient’s EGD was normal aside from one benign fundic gland polyp. According to the proposed diagnostic criteria by Worthley et al. [1], the woman’s endoscopic findings would not be diagnostic of GAPPS. The occurrence of reduced penetrance or just variable expression in GAPPS has been reported previously [1, 2, 5] and is likely due to a complex interaction of genetics, environmental, and lifestyle factors. Aside from the woman’s medical history, she also had multiple diagnoses of cancer. This was also observed in the mother in Family I, whose endoscopic findings were consistent with those previously described for GAPPS. We did not observe any difference in penetrance regarding gender, which is consistent with previous reports, nor did we observe any age‐dependent penetrance. Nevertheless, the variable phenotype complicates genetic counseling of these patients even further. As GAPPS predisposes to gastric cancer, the risk with abstaining from genetic testing or the abruption of endoscopic surveillance due to a lacking phenotype could potentially be fatal. On the other hand, routine endoscopies can be debilitating and give rise to unnecessary worries. More studies, preferably with a longer follow‐up period, need to be performed to determine the properties of penetrance in relation to GAPPS.

Furthermore, we observed a difference in the dysplasia status amongst the family members, with some having multiple areas of dysplasia and others having no areas of dysplasia. The status of dysplasia did not seem to correlate with age, as several of the children from Family I had low‐grade dysplasia, while their grandmother did not. The variable expression in dysplasia status could be due to the limitations of endoscopic surveillance in patients with GAPPS. As many patients have extensive polyposis (“carpet‐like” involvement) in the gastric area mixed with adenomas and hyperproliferative pits, it becomes more challenging to collect the right sample biopsies reflective of the underlying status of dysplasia. Due to the extensive polyposis, there is a risk of potentially missing areas of focal progression and malignant transformation. The histopathological findings after gastrectomy in three of the family members showed dysplasia in all three, whereas only two of them had dysplasia prior to surgery. This raises the question of endoscopic surveillance versus prophylactic gastrectomy in this patient group. The decision on whether to perform prophylactic gastrectomy and the timing of the operation must be carefully considered, as an increased risk of morbidity and mortality postgastrectomy is still observed [11]. The Danish Surgical Society and the Danish Society of Medical Genetics have developed a guideline in 2021 for GAPPS [12]. They recommend upper endoscopic surveillance annually in patients from the age of 15 and eventually more frequently if polyps are present. Prophylactic gastrectomy is recommended if dysplasia is identified in the biopsy specimens. However, these recommendations are solely based on expert opinion.

Our results also indicate that patients with GAPPS do not have a colorectal FAP phenotype. Thus, colonoscopy was performed on all the patients from both families, only detecting five hyperplastic polyps, seven adenomas, and one sessile serrated lesion divided amongst four family members. The detected colorectal polyps were primarily found in the older family members, aged > 60 years, and could simply be reflective of sporadic colorectal polyps, which are frequently seen in the background population [13]. Therefore, our data cannot support FAP surveillance of the lower GI tract.

The genetic cause of GAPPS is a pathogenic variant in the promotor 1B region of the APC gene. Consequently, a variant can easily be overlooked when using restricted gene panels. The detection of a variant in the promotor region requires next generation sequencing (NGS), such as whole genome sequencing (WGS) or gene panels that include the promotor 1B region.

In conclusion, we present the first eight Danish patients with GAPPS. Our report highlights the variable phenotypic expression in GAPPS and, consequently, the difficulty in surveillance and genetic counseling of these patients and their families.

## Ethics Statement

Written informed consent was obtained from all the included patients.

## Conflicts of Interest

The authors declare no conflicts of interest.

## Author Contributions

P.A.S‐R., A.M.J., and T.D.J. designed the case report. P.A.S‐R. drafted the manuscript. A.M.J. and T.D.J. included the patients for the case report. P.A.S‐R., A.M.J., J.G.K., A.H.P., M.B.M., and T.D.J. critically revised and finally approved the manuscript.

## Funding

This research received no specific grant from any funding agency in public, commercial, or not‐for‐profit sectors.

## Supporting Information

Video 1. Esophagogastroduodenoscopy of a 28‐year‐old GAPPS patient seen at Hvidovre Hospital, Denmark. The EGD shows “carpet‐like” fundic gland polyposis of the stomach with sparring of the antrum.

Supporting Information 1. Individual patient data including age, gender, variant, gastric manifestations, colorectal manifestations, helicobacter pylori status, mortality, and information on gastrectomy, gastric cancer, and colorectal cancer.

## Supporting information


**Supporting Information** Additional supporting information can be found online in the Supporting Information section.

## Data Availability

All data generated or analyzed are included within the article.
